# Discordance, Disclosure and Normative Gender Roles: Barriers to Couple Testing Within a Community-Level HIV Self-Testing Intervention in Urban Blantyre, Malawi

**DOI:** 10.1007/s10461-018-2038-0

**Published:** 2018-02-06

**Authors:** Moses Kelly Kumwenda, Elizabeth Lucy Corbett, Jeremiah Chikovore, Mackwellings Phiri, Daniel Mwale, Augustine Talumba Choko, Marriot Nliwasa, Rodrick Sambakunsi, Miriam Taegtmeyer, Tore Jarl Gutteberg, Alister Munthali, Nicola Desmond

**Affiliations:** 1 Behaviour and Health Group, Malawi Liverpool Wellcome Trust, Blantyre 3, P.O. Box 30096, Chichiri, Malawi; 2 College of Medicine, Helse Nord TB Initiative, Blantyre 3, Private Bag 360, Chichiri, Malawi; 3 Department of Clinical Research, London School of Hygiene and Tropical Medicine, Keppel Street, London, WC1E 7HT UK; 4 HIV/AIDS, STIs and TB (HAST), Human Sciences Research Council, Private Bag X07, Dalbridge, 4014 South Africa; 5 International Public Health, Liverpool School of Tropical Medicine, Pembroke Place, Liverpool, L3 5QA UK; 6 Department of Medical Biology, University of Tromsø, Postboks 6050, Langnes 9037, Tromsø, Norway; 7 Department of Microbiology and Infection Control, University Hospital, UNN Tromsø, Klinikk/avdeling, 9038 Tromsø, Norway; 8 Centre for Social Research, Chancellor College, P.O. Box 280, Zomba, Malawi

**Keywords:** Disclosure, Discordance, Gender, Couple, Self-test

## Abstract

A community-based HIV self-testing study in Blantyre, Malawi demonstrated that not all individuals living in couples tested with their partner. We describe factors dissuading individuals in couples from self-testing with their partner. Data were drawn from qualitative study exploring consequences of HIV self-testing within couples. In-depth interviews were conducted with 33 individuals living in couples who tested alone. Participants expressed fear of dealing with HIV-discordant relationships. Failure to self-test with a partner was correlated with gender, with more men than women overtly declining or unconsciously unable to have joint HIV self-test. Men feared exposure of infidelity and were often not available at home for economic reasons. Barriers to uptake of couple HIV self-testing seemed to be shaped by gendered dichotomies of social-relationships. To help achieve the first 90% of the UNAIDS 90:90:90 goals, it is important to overcome structural barriers to realise the full potential of HIV self-testing.

## Introduction

In 2012, 2.3 million people were infected with HIV globally and 70% of all new HIV infections occurred in sub-Saharan Africa [1]. In this global epicentre, nearly two-thirds of all new HIV infections occur within established heterosexual relationships due to pre-existing discordance—where one partner is HIV-positive and one HIV-negative—or because of extra-marital exposure [2]. In sub-Saharan Africa, up to half of all HIV-infected people living in established heterosexual relationships are HIV-discordant [3, 4], with recent data showing a conversion rate of 1 in 5 annually [5]. Both men and women have equal chances of being the index partner in a discordant relationship [6]. Only half of men and women living with HIV (15 years and older) in sub-Saharan Africa know their status [1] and this contributes to ongoing transmission of HIV at the community level.

Modelling data predict that HIV testing and counselling which target couples (CHTC) has the potential to avert HIV transmission from 7 to 20% annually, thus preventing up to 60.3% of new HIV infections [7], largely due to increased levels of disclosure. Additional benefits of CHTC include increased uptake and adherence to PMTCT; safer contraception; increased uptake and adherence to ART; increased uptake of voluntary medical male circumcision (VMMC); decreased stigma; and HIV prevention to external partners [8, 9]. The current HIV testing trends illustrate higher rates of testing amongst women, largely through antenatal care (ANC) HIV testing services [10], and lower rates of HIV-testing rates amongst men [11] and even suboptimal uptake of CHTC [12, 13].

HIV self-testing (HIVST) is a novel HIV screening approach that involves an individual performing an HIV test and interpreting the test results in private [8] and has great potential for increasing the uptake of couples testing and reaching male partners [14, 15]. A recent couple-targeted clinical trial in Kisumu, Kenya demonstrated a high uptake of couple testing achieved through the use of oral-fluid-based HIV test kits delivered through antenatal and postpartum women [16]. However, unpublished data from the second round of implementation of community-level HIVST in Blantyre, Malawi demonstrated that only 16%^1^ of HIV self-tested individuals (1205 out of 15,106) tested as couples despite an offer of an extra test kit for the partner [17]. Here, we describe discordance, disclosure and gendered unavailability of male partners as important factors that dissuaded partners from self-testing together within a context of limited HIV testing options for couples.

## Theoretical Framework

We used sociological perspectives of gender, emphasising the socio-structural determinants of gender differences as a theoretical framework [18, 19]. Social structural theory considers sexual differences to originate from the contrasting social positions of women and men and maintains that gender differences are socially constructed rather than inborn, since institutional and social practices shape gender differences [20]. Thus, the normative gender stereotypes determine how men and women are perceived, evaluated and treated [21]. Social norms delineate men from women by assigning different behaviours to them and attaching different meanings to their actions.

## Methods

This study was nested in a cluster randomized trial (CRT) investigating the impact of intensified HIV/TB prevention which implemented community-level HIVST in urban Blantyre [17]. Our previous paper described how the community counsellors (CC) provided HIVST to community members through a community-based model and how participants of this study were recruited [15]. This study recruited participants who self-tested within the CRT and who had no prior knowledge of the interviewers before commencement of data collection. Data were collected between October 2012 and February 2014.

In-depth interviews (IDIs) were conducted within a month of self-testing with 33 individuals living in established heterosexual relationships who tested without a sexual partner. The decision to use qualitative methods allowed for a deeper exploration of social issues within the specific context. The IDI data collection strategy used also allowed a deeper understanding of normative relationship dynamics generated from personal accounts [22] of self-testers. The interactive nature of IDIs provided study participants an opportunity to talk about what they felt comfortable with and then allowed interviewers to use a range of probing questions to achieve more in-depth responses [22] and to clarify emerging issues.

We deliberately varied the demographic attributes of participants by gender, HIV sero-status and knowledge of partners status using maximum variation approach [23] in order to have a broader distribution of participant types to maximize representation in each selected category (see Table 1).

**Table 1:** Purposive sampling framework of individuals self-testing without a partner

Sex	Participant sero-status	Number recruited
Male	HIV-positive	4
	HIV-negative	5
	HIV-positive or negative but unaware of partners status	4
Female	HIV-positive	7
	HIV-negative	7
	HIV-positive or negative but unaware of partners status	6

^It was difficult to balance male and female study participants in the purposive sample because men were difficult to find especially HIV-positive me^

Our sample had more female study participants (n = 20) than males (n = 13). The difference in male and female participation rates occurred because certain groups of participants were difficult to find during recruitment, including men, HIV-positive people and people in HIV-discordant relationships. The ages of the study participants ranged between 18 and 53 years old, with males being slightly older (age range between 20 and 53) than females (age range 18 to 42). Male participants were better educated (i.e. primary level = 4; secondary level = 8; tertiary level = 1) than female (i.e. primary level = 11; secondary level = 8; tertiary level = 1). Male participants were also better employed (i.e. all 13 participants) than females (i.e. 4 out of 20), but most of their jobs were in the informal employment sector.

Data were gathered by experienced qualitative researchers Moses Kumwenda (MK), Mackwellings Phiri (MP) and Daniel Mwale (DM) within a month of self-testing in order to improve recall of key issues. A pre-tested topic guide was used to structure the interviews which lasted between 25 minutes and 1 hour while digital audio recorders captured the conversation in Chichewa, the dominant local language. Interviews explored reasons for testing, enablers and barriers to partner testing, and reflections on having self-tested. Summative field notes were made during and immediately after the interviews to provide quick impressions of the emerging themes. Recorded audio data were then transcribed verbatim, cleaned and reviewed for accuracy. Sexual partners were interviewed separately to overcome methodological and ethical challenges linked to recruitment, consenting partners, and confidentiality [24], and other adult non-study participants were not allowed to be present during the interviews.

Transcripts were imported into NVIVO 9 QSR software (QSR, Melbourne, Australia) for organizing, managing and coding. Units of emerging themes for analysis were coded from Chichewa transcripts by three Chichewa speakers (MK,MP and DM) to optimise trustworthiness of interpretation and credibility while presentation of the results was guided by COREQ guidelines [25]. During data collection and analysis, it was evident that the data set was complete and a saturation of information was reached, indicated by data replication or redundancy [23, 26]. Data were triangulated across different gender and sero-status groups of participants [27] to ensure validity. Transcripts were read, re-read, coded and classified using content analysis [28, 29] by MK, MP and DM. Similar units of categories were then combined to generate broader themes and presented as descriptive narratives.

The study was approved by the College of Medicine Research Ethics Committee (COMREC), affiliated with the University of Malawi, and all the study participants provided written informed consent.

## Results

**Fig. 1:**
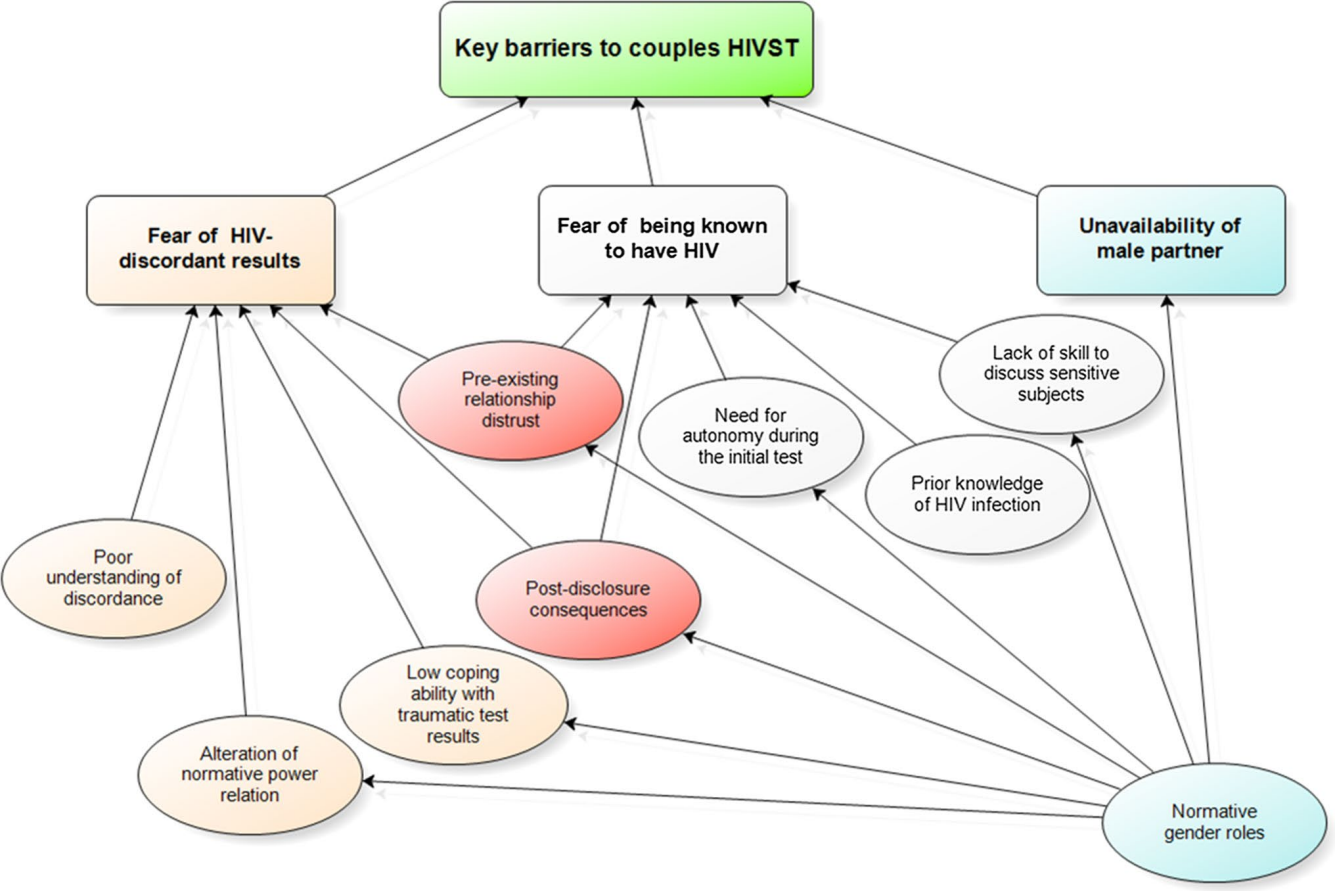
Emerging themes on key barriers to couples HIVST

A range of issues prevented couples self-testing together. We present three main themes, namely: the fear of dealing with being in an HIV-discordant relationship, the fear of having to disclose a possible HIV-positive status and the gendered unavailability of male partners at home, with additional sub-themes feeding into these three main themes as summarised in Fig. 1.

### Fear of Dealing with an HIV-Discordant Relationship

Men and women were afraid of the disclosure of HIV-discordant relationships and the potential negative consequences of this test result on their relationships, which were based on trust. Our data showed that participants did not understand what HIV-discordance was and how it occurred in trust relationships characterised by unprotected sex. In addition, study participants did not know what sexual partners should do after HIV-discordant results were revealed, and many considered divorce as the only solution. Thus, partners in a relationship with a previous history of confirmed or suspected infidelity were afraid to self-test together fearing that one partner might test HIV-positive. Among the perceived harmful effects of being in an HIV-discordant relationship (as cited by the study participants) are physical or psychological violence, separation, deprivation of unprotected sex and divorce:

…when the result shows that your partner has been found with the virus and you have not been found with the disease, ‘can the marriage continue?’ No, it can end because the uninfected partner would be afraid of getting infected. This is what frustrated me from testing with my wife…. (Male, 27 years, HIV-positive, concordant, secondary education, informal employment)

A positive HIVST result for one partner was said to invite unfavourable dynamics within the relationship often carried out by the HIV-uninfected partner. In terms of effects on the sexual relationship the uninfected partner was usually worried about contracting HIV while the infected partner was afraid of being denied access to unprotected sex. Men and women seemed to anticipate the social effects linked to an HIV-discordant relationship differently. The fears of marriage dissolution following HIV-discordant results were more profound amongst women as they were primarily worried that a positive result would jeopardise financial support for themselves and their children and severely damage their respectability in the community:

…if I am found with it [HIV] while my husband doesn’t have it, he would consider me as a prostitute and may choose to chase me away [divorce]. (Female, 29 years, HIV-positive, concordant, primary education, unemployed)

Men were mostly worried that a positive result would undermine their masculine authority in the household and were afraid to relinquish control over unprotected sex, as described by a woman in this quote:

He was afraid that if we test together and our results are HIV-positive, the counsellor would instruct us to use ‘zishango’ [condoms] which he does not like to use. (Female, 27 years, HIV-positive, discordant, secondary education, formal employment)

### Fear of Exposure of a Possible HIV-Positive Result and Linked Negative Consequences

Fear of exposing a possible or in some cases an already known HIV-positive result to a partner through joint testing emerged as a common theme among individuals who self-tested in absence of the sexual partner. For some men who tested alone, the main reason their female partner refused couples testing was not being prepared to have her HIV-positive status known by her partner through a joint HIVST. Declining to self-test with a partner was common among some women who already knew their HIV-positive status but were unprepared to disclose it to a male partner, as recalled by a man whose partner refused a joint HIVST:

My wife was also at home (when HIVST was offered through door-to-door approach). She refused because she was not ready to test at that time. (Male, 31 years, HIV-positive, concordant, secondary education, informal employment)

The man also indicated that his partner seemed to have been aware of her HIV-positive status but did not disclose it out of fear of possible marriage dissolution.

In contrast, women who self-tested alone indicated that their male partners frequently refused a joint HIVST because they feared having an initial HIV test in the presence of a partner. Men feared blame from a partner for introducing HIV into the relationship. Some men even issued strong threats to their partner to intimidate them from pursuing the joint HIVST agenda as illustrated in a quote by an unemployed female partner recalling how her male partner refused an offer of a joint HIVST:

‘I don’t want. I cannot test, no. I will go to a clinic myself to test. What have you seen in me?’ He also threatened me that ‘if you test, we will see what will happen to you inside the house’. (Female, 29 years, HIV-positive, concordant, primary education, unemployed)

In this example, the male partner, who later also self-tested HIV-positive, used his authority to suppress and discourage his partner’s request for a joint HIVST, a theme that recurred in most of the interviews. Men’s position of power within the household gave them greater leverage to obstruct or neutralize any suggestion to undergo a joint HIVST coming from their partner.

Men who felt they were distrusted by their partner opted for an initial individual self-test to preserve their ability to conceal their status in the event that the test-results came back positive and thus be able to maintain their moral credibility. In short, many participants seemed uncomfortable and/or lacked the skills to openly discuss these issues with their partner. Within the context of established couples, HIV-positive results are usually synonymous with previous or current infidelity, as one man explained:

…should I test together with my wife, no….if she knows my status, she might be think[ing] that I sleep around with other women. It is better for me to test alone. (Male, 20 years, HIV-negative, partner sero-status unknown, primary education, informal employment)

Thus, men declined joint testing in order to safeguard trust within the relationship. This demonstrates that men have a higher perception of subjective risk, leading to fears of testing for HIV on the one hand and of disclosing their HIV positive sero-status on the other. Men were much more likely than women to fear self-testing together with their partner due to a previous or current record of infidelity as illustrated by this quote:

I had fear after doing something [engaging in sexual misconduct]. If I say something, you know what I mean…. If I have slept [had sex] with other women… I was afraid of considering that the truck [him as a man] had travelled a long distance and with heavy load of goods to reach the destination to deliver the goods. (Male, 32 years, HIV-negative, discordant, tertiary education, formal employment)

In this quote, the word ‘truck’ was a metaphor for ‘man,’ the ‘load of goods’ represented the number of women that he had had sex with, and the ‘distance to the destination’ referred to the time from the onset of being sexually active. The partner’s suspicions of sexual malpractice amplified the man’s fear of the consequences following self-testing in the presence of his partner. Apart from exposing concealed infidelity, male participants were also worried that an HIV diagnosis through a joint HIVST would render them vulnerable to blame and accusation for introducing HIV into the relationship, as illustrated in this quote.

When you have been stepping your bare feet on thorns [having risky sexual affairs], it makes you feel that you have injured [infected] your friend [sexual partner]. I avoided testing together with her because I felt that she would blame me. (Male, 32 years, HIV-positive, concordant, secondary education, informal employment)

In the quote above, stepping on thorns refers to engaging in unprotected sexual encounters. The quote illustrates a feeling of not wanting to hurt a sexual partner but also a degree of cognitive dissonance between behaviour and intention. While men were worried about blame following HIV status disclosure, women feared being branded as unfaithful and a possible domestic violence for requesting a joint HIVST.

### Gendered Unavailability

The absence of one partner at home at a time of a CC’s door-to-door visit deterred some couples from having a joint HIVST. While this reason was provided by both men and women, it was especially frequent among women who self-tested alone because their partner was not at home. Since HIVST was mostly offered during the normal working hours, the CCs found women at home more often, as men had gone to work. Women commonly stated that they self-tested without a partner because the male partner was at work when HIVST was offered. This quote speaks to the unavailability of the male partner:

… he was at work… I self-tested alone because it is me who has this body and it is me who feels the pain. Because of this, they say ‘a bag of life is cared by oneself’. (Female, 41 years, HIV-positive, concordant, primary education, unemployed)

The quote illustrates to some degree that people act in their own interest where an individual cannot expect support from his/her partner, based on a premise that the couple is in this together. It also demonstrates the potential to be blamed for the inability to care for one’s own state of health. A metaphor ‘bag of life’ was used in this context to demonstrate that safeguarding one’s health is one’s own responsibility. Men who self-tested as individuals mostly cited that they tested alone because their female partner had either gone to the market, to the hospital or to the village at the time HIVST was offered. The places mentioned here correspond with the normative gender roles ascribed to women in the study setting:

… at that time, my wife had gone to under-five clinic with a child. …I thought that ‘since the person [CC] is already here, I should just self-test. (Male, 31 years, HIV-positive, concordant, secondary education, informal employment)

The masculine provider role meant that many men were away from home while the feminine caregiving role kept most women at home and increased their likelihood of being reached by HIVST. Despite the fact that the community-based HIVST model appealed to both men and women, its design rendered HIV testing more accessible to women than men, thereby reproducing the current HIV-testing variations.

## Discussion

**Fig. 2:**
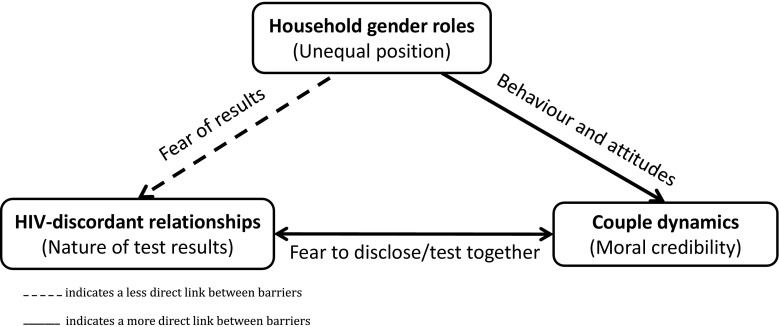
Intersection of the key barriers to couples HIVST

Our study has shown three key but related barriers for couples to self-testing together within a community-based HIVST model in urban Blantyre (see Fig. 2). The study is the first to highlight the fear of being in an HIV-discordant relationship as a barrier to couples testing. The fear of a partner knowing one’s positive HIV status and the unavailability of men when HIVST is offered have been shown as two other important barriers to couples self-testing together. These less commonly expressed barriers to couples testing occur despite the greater acceptability of HIVST and the high uptake of the self-testing approach by both men and women [17].

The fear of HIV-discordant and HIV-positive concordant results was also reported as barriers to CHTC in Uganda, but with inconclusive evidence as to which of the two was most feared in the context of established sexual relationships [30]. Some studies have generally shown the fear of HIV-positive results as the primary barrier to CHTC [30–32]. Here, we have highlighted that HIV discordance is more feared because discordant results present difficult and complex dynamics which partners are less equipped to manage [33]. Being in a trust relationship and the general understanding that HIV is largely transmitted through unsafe heterosexual contacts meant that discordant results after CHT exposed the infected partner as being disloyal by engaging in extra-marital sex and careless for not using protection. This fear echoes findings from Zambia which demonstrated that marital partners feared the psychological consequences when a partner learns one’s HIV status and the corresponding need to protect one’s personal image of moral credibility [34]. The fear of blame for bringing HIV into the relationship and potential exposure of concealed extra-marital relationships reported in this study also prevented the uptake CHTC in Uganda [30].

The normative gender roles, where husbands are expected to provide while wives take care of the children at home, meant that the community-based approach found women at home much more often when HIVST was offered through door-to-door approach. The backdrop of the disempowered socio-economic position of women advantaged them to have easy access to HIVST since unemployment meant they were usually found at home. A study in Uganda reported that the conflicting work schedules between male and female sexual partners made one of the partners, particularly men, not available to receive HTC together with their partners [30]. Men are not found at home because their role as household providers require them to go to work and work hard even at the expense of their own health in order to support the immediate and extended families [35, 36]. The traditional gender roles meant that men spent more time at work than home to fulfil their provider roles, and hence were often missed by a community-based HIVST strategy [37–40]. Thus, gender seemed to influence the politics of established sexual relationships by determining partners attitudes towards an offer of a joint HIVST [41, 42].

These results emphasise that couples should be targeted by more flexible HIVST models that consider the needs of male partners in urban settings in order to increase rates of partner notification, reduce HIV transmission, promote early uptake of treatment and foster treatment adherence. Results suggest the importance of demystifying HIV-discordance in the community, especially within the context of HIVST, by explaining the benefits of knowing HIV-discordant status and focusing on protective measures and supporting couples testing. Our results also speak to the need for proper training of community-level HIVST providers and counsellors regarding information given to clients.

Although HIVST is an acceptable and feasible approach for reaching male partners, these results may be necessary for refining the current community-based delivery models to improve their responsiveness to the needs of couples [17, 43–45]. This includes identifying strategies to optimise access to HIVST among male partners, often underserved because of their normative gender roles, by exploiting the convenience, empowering and assurance attributes of HIVST. This strategy should be implemented whilst critically revisiting the role that gender plays in dissuading couples from testing together as couples. Such an approach could be very useful especially where the female partner has diminished ability to singlehandedly convince the male partner to test for HIV. These findings have implications for programmes but also for counsellors who see/support individual self-testers coming for confirmatory testing.

## Limitations

Our analysis was conducted within the context of a CRT implementing HIVST in a manner that is unlikely to be adhered to outside of the research context and that included door-to-door promotion of HIVST as well as self-presentation of clients. Participants often confused discussions of HIVST as being identical to discussions around door-to-door access (which has high uptake and tends to be favoured over facility-based testing even when providing conventional HTC and not HIVST). Lastly, the use of an all-male data collection team may have influenced the quality of data collected especially from female participants. However, the vast experience of the team members in doing qualitative researcher and occasional support from a female qualitative researcher/transcriber helped overcome this challenge.

## Conclusion

Barriers to uptake of couple HIVST for individuals living in established relationships seemed to be shaped by the gendered differences of social-relationships which are clearly visible within the contemporary societies in urban Blantyre, Malawi. When faced with an opportunity to access couples HIVST, the fear of HIV-discordant relationships moderated by structural gender differentiation influenced how individual partners navigated and negotiated their decisions to either accept or decline self-testing together with a partner. Burdened by the role as household providers, men were often unavailable for HIV self-testing provided in the community and the opportunity to test together with their partner. Furthermore, the social positions of men and women shaped by the normative gender roles and gender stereotypes made it difficult for men to access HIVST delivered through a community-based approach. In order to contribute towards achieving the first 90% of the UNAIDS 90:90:90 goals, it is important to overcome barriers to couples HIV testing that limit the realisation of the full potential of HIVST in underserved groups of people such as couples. Re-examining the social context and evaluating the impact of pre-existing gender norms may be useful to align and optimise community-based HIVST models to the specific needs of couples living in HIV-endemic settings.

## Footnotes

1 This estimate may be lower than the actual estimate for couples HIVST since 15,106 is not a true denominator, it include even those who were not in partnerships.
